# I remember you! Multicomponent warning signals and predator memory

**DOI:** 10.1093/beheco/arae092

**Published:** 2024-11-19

**Authors:** Anita Szabó, Magdalena Bělová, Alice Exnerová

**Affiliations:** Department of Zoology, Faculty of Science, Charles University, Viničná 1594/7, Prague, 12844, Czech Republic; Department of Zoology, Faculty of Science, Charles University, Viničná 1594/7, Prague, 12844, Czech Republic; Department of Zoology, Faculty of Science, Charles University, Viničná 1594/7, Prague, 12844, Czech Republic

**Keywords:** avian predators, color, discrimination learning, long-term memory, multicomponent signals, pattern, warning coloration

## Abstract

To avoid potentially noxious prey, predators need to discriminate between palatable and unpalatable prey species. Unpalatable prey often exhibits visual warning signals, which can consist of multiple components, such as color and pattern. Although the role of particular components of visual warning signals in predator discrimination learning has been intensively studied, the importance of different components for predator memory is considerably less understood. In this study, we tested adult wild-caught great tits (*Parus major*) to find out, which components of prey visual warning signals are important when the birds learn to discriminate between palatable and unpalatable prey, and when they remember their experience over a longer time period. Birds were trained to discriminate between palatable and unpalatable artificial prey items that differed in both color and pattern. After 4 wk, the birds were retested in 3 groups: the first group was presented with the same prey as in the training, the second group was tested with the two prey types differing only in color, and the third group could use only the pattern as a discrimination trait. The results suggest that the birds remember their experience with unpalatable prey even after the period of 4 wk. Although the color appears to be more important than the pattern, the combination of both signal components is more effective for prey recognition after several weeks than either the color or pattern alone.

## Teaser

Unpalatable prey species usually exhibit warning signals, which—if recognized by predators—can increase the survival of prey. Using an experimental approach, we show that great tits (*Parus major*) remember their experience with unpalatable prey for the period of 4 wk after the initial encounter. While doing so, they attend to the color of the prey first and then to the pattern.

## Introduction

To ward off potential predators, unpalatable prey often uses conspicuous visual warning signals (Ruxton et al. 2018). Several studies have shown that the typical warning coloration, such as red or yellow combined with contrasting black pattern, makes the aposematic prey distinct from the undefended palatable prey and conspicuous against natural backgrounds (Sherratt and Beatty 2003; Penacchio et al. 2024). This helps predators recognize unpalatable prey more easily (Guilford 1986; Gamberale-Stille 2001; Hunter 2009) and enhances discrimination learning (Aronsson and Gamberale-Stille 2009; Rönkä et al. 2018 but see; Ham et al. 2006) and prey memorability (Johnston and Burne 2008; Ko et al. 2020). The conspicuousness of prey coloration is also important for prey generalization (Gamberale-Stille and Tullberg 1999). For example, Svádová et al. (2009) showed that great tits (*Parus major*) did not generalize from red firebugs (*Pyrrhocoris apterus*) to yellow and white morphs, whereas birds that learned to avoid yellow color morph generalized their experience toward the more conspicuous red morph. In contrast to this, Rönkä et al. (2018) did not find any evidence for generalization between different color morphs of the wood tiger moth (*Arctia plantaginis*) in blue tits (*Cyanistes caeruleus*).

Visual warning signals are often multicomponent, that is, they consist of several traits such as color, pattern arrangement, inner pattern contrast, background contrast, pattern symmetry, and body shape (Rowe 1999; Aronsson and Gamberale-Stille 2012). Different signal components may be important in defense against multiple predators that differ in their visual perception. For instance, color appears to be the most salient trait in prey discrimination learning for tetrachromatic avian predators (Aronsson and Gamberale-Stille 2008), whereas luminance contrast plays an important role in avoidance learning of color-blind mantises (Prudic et al. 2007). At the same time, different signal components may act synergistically and affect the response of one and the same predator (Rowe 1999; Sherratt and Holen 2018; Kikuchi et al. 2023).

Several studies have shown that birds primarily attend to color during prey discrimination learning and generalization (Aronsson and Gamberale-Stille 2008; Kazemi et al. 2014) and that they also exhibit accurate memory of colors (Aoki et al. 2000). Moreover, color may completely overshadow other visual traits, such as contrasting patterns as potential prey discrimination cues even if other traits are equally informative about prey palatability (Aronsson and Gamberale-Stille 2008).

Although color appears to be the most important discrimination cue for birds, in cases when color is not a reliable predictor of prey palatability or when other traits, such as pattern convey additional information, birds can use other visual traits for prey discrimination in a hierarchical manner (Aronsson and Gamberale-Stille 2012). For example, great tits (*P. major*) attend to internal pattern and body shape when discriminating between different species of ladybirds (Dolenska et al. 2009). The presence of black pattern also increases both the internal contrast and contrast against the background and may thus contribute to faster avoidance learning (Aronsson and Gamberale-Stille 2009, 2013) and easier recognition of aposematic prey (Gamberale-Stille 2001; Sherratt and Beatty 2003). In contrast, pattern regularity and symmetry do not seem to further enhance discrimination learning (Aronsson and Gamberale-Stille 2013).

Different visual signal components could also differ in how they interact with signals from other modalities as a part of multimodal signals (Rowe and Guilford 1999; Rowe and Halpin 2013). For instance, Hauglund et al. (2006) found that for domestic chicks (*Gallus domesticus*), yellow color was the most effective in triggering innate biases, whereas the presence of stripes contributed to faster avoidance learning and slower memory extinction rates. Furthermore, they found that sound (i.e. buzzing) did not contribute to innate aversion but increased the effect of stripes during the avoidance learning in case of the green prey. Although the role of particular components of visual warning signals in predator discrimination learning has been intensively studied, the importance of different components for predator memory is considerably less understood.

Predators may benefit from remembering their experience with prey over a long time period, for instance, in temperate habitats, where prey abundance fluctuates between seasons, and predators might not encounter most prey species during the winter. Thus, predators frequently need to retain their memory of prey appearance over several weeks or months. To date, only a few studies have investigated long-term memory retention of prey warning signals by predators. Domestic chicks (*Gallus domesticus*) were shown to avoid unpalatable prey with typical warning coloration (black and yellow stripes) 24 h after the discrimination training (Johnston and Burne 2008), and increased unpalatability of prey contributed to better memory retention in chicks after a period of 48 h (Skelhorn and Rowe 2006). Agamid lizards (*Diploderma swinhonis*) were shown to remember aposematic prey coloration 60 d after a single aversive experience (Ko et al. 2020), and jungle crows (*Corvus macrorhynchos*) retained their memory of rewarded colors for 10 mo after the discrimination training (Bogale et al. 2012). However, studies investigating the role of different components of visual warning signals in predator memory are lacking.

Our aim was to assess the importance of the 2 key components of visual warning signals, color, and pattern, in the long-term memory retention following the prey discrimination training, and how well the birds remember their experience after 4 wk without any contact with the discriminated prey types. Because the previous studies have shown that birds primarily attend to color when discriminating between palatable and unpalatable prey (Aronsson and Gamberale-Stille 2008, 2012), we predicted that color would be a more important cue than pattern for the long-term memory retention. We tested adult wild-caught great tits (*P. major*) first in a discrimination learning task and subsequently in 2 memory tests using palatable and unpalatable artificial prey items printed with the stimuli differing in color and/or pattern. We used cyan and magenta colors, which do not usually occur in warning signals in nature in order to minimize potential biases caused by previous experience of birds with defended prey from the wild.

## Material and methods

### Birds

Altogether, we tested 41 wild-caught great tits *(P. major* L. 1758) during the years 2017 to 2020 (6 birds in 2017, 4 birds in 2018, 19 birds in 2019, and 12 birds in 2020). Birds were caught using mist nets in the Botanical garden of Charles University in Prague (50.08 N, 14.24 E, Czech Republic) over the year except for the breeding season (March to July). Birds were sexed and aged upon capture differentiating between 2 age categories: yearlings and older adults (Svensson 1992). We tested 20 males and 21 females and 21 yearlings and 20 older adults. After catching, the birds were allowed to habituate to their home cages (50 × 40 × 50 cm) for 2 to 5 d before the beginning of the experiment. Birds were housed individually but had visual and acoustic contact with other birds in the aviary. Light conditions were set to correspond to the natural photoperiod. Birds were offered a diet consisting of mealworms (larvae of *Tenebrio molitor*), sunflower seeds, and commercial food mixtures for insectivorous birds (Uni Patee and Insect Patee, Orlux).

### Artificial prey

**Fig. 1.**
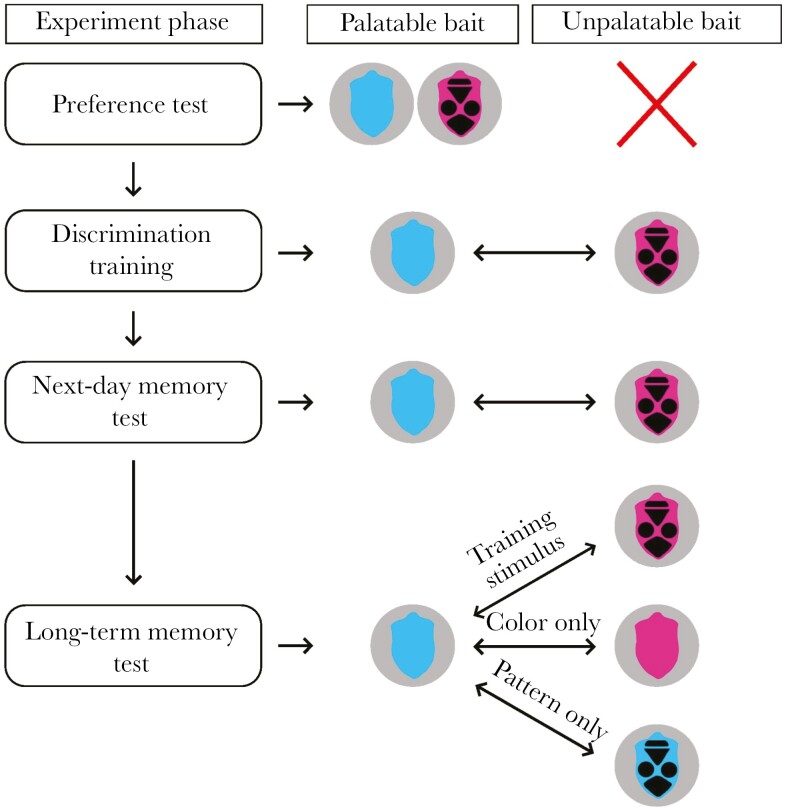
Experimental design and the set of artificial prey items used in different phases of the experiment.

As prey items, we used paper baits (15 × 10 mm) with a piece of mealworm underneath. The shape of the baits was based on the real insect prey (shield bugs, Heteroptera: Pentatomidae). Paper baits were printed with either cyan (Hex-code: 02c0f7 – hue: 193, saturation: 99%, lightness: 97%, RGB: 2, 192, 247) or magenta (Hex-code: f604b4 – hue: 316, saturation: 98%, lightness: 96%, RGB: 246, 4, 180) color and either black pattern or not depending on the treatment (Fig. 1). The cyan and magenta colors were selected to minimize the potential biases caused by the birds’ previous experience with insects from the wild. The shape, colors, and pattern of the baits were designed in Adobe Photoshop, and then printed (Xerox Versant printer; color profile FOGRA 39) on a white 350 g/m^2^ cardboard paper. The pieces of mealworms were either soaked in water (palatable prey) or in a 6% quinine-phosphate solution (unpalatable prey) before each trial. Quinine is an odorless substance with an intensively bitter taste and makes the food aversive for birds (Lindström et al. 1999a, Ham et al. 2006). Birds were not allowed to see the pieces of mealworms, only the color and pattern of the paper baits. The prey items were presented to the birds in glass Petri dishes against an achromatic gray (lightness: 40%, RGB: 102, 102, 102) background.

### Experimental setup

Experiments were conducted in wooden cages (70 × 70 × 70 cm) with wire-mesh side walls and a front wall made of 1-way glass through which the birds were observed. The cages contained a wooden perch, a water bowl and a rotating wooden tray with 6 circular cups. The cages were illuminated by Biolux Combi 18W (Osram) bulbs to simulate natural light conditions. Before each experiment, birds were deprived of food for 2 h to increase their foraging motivation. Water was accessible ad-libitum throughout all the experiments.

### Experimental design

Prior to the experiment, birds were allowed to habituate to the experimental cage and pre-trained to search for pieces of mealworms placed underneath a white paper square (2 × 2 cm) in one of the cups. The pre-training was considered successful when the birds learned to turn the paper square and ate the piece of mealworm stuck underneath at least 3 times in a row.

After the pre-training, each bird took part in a preference test to assess initial preferences for one of the prey types. In each of the 5 consecutive 4-min trials, birds were offered two paper baits simultaneously: cyan without pattern and magenta with a black pattern (Fig. 1), both baited with a palatable piece of mealworm. The order of handling the two prey types was recorded. Handling was defined as touching the prey with the beak, which did not necessarily include tasting the mealworm. In order to complete the preference test, birds had to taste both prey items at least once during the 5 trials otherwise the last trial was repeated.

The next day, birds participated in the discrimination training, which consisted of 40 trials, each lasting for 4 min. During this phase, all birds were trained to discriminate between palatable and unpalatable prey items based on both the color and pattern of the paper baits. Palatable prey items were associated with plain cyan-colored paper baits and unpalatable prey with magenta-colored baits bearing black pattern (Fig. 1). This way, birds could use both the bait color and presence/absence of the black pattern as a discriminative cue. Both prey items were offered simultaneously in a Petri dish placed in one cup of the feeding tray, and their positions were alternating in each trial to prevent birds from discriminating between palatable and unpalatable prey items according to their positions. To get information about prey palatability, birds had to taste both prey items at least once during the first 5 trials, otherwise the trials were repeated. In each trial, we recorded whether the bird handled one or both prey items, which prey item was handled first, latency of handling, and whether the bird ate (or attempted to eat) the pieces of mealworms. Again, handling was defined as touching the prey with the beak, which did not necessarily include tasting the mealworm. If the birds did not touch any of the prey items during a trial, it was repeated after 5 to 10 min.

The next day, birds were subjected to the first memory test to assess if they remembered their experience from the discrimination training. The next-day memory test consisted of 10 trials with the same paper baits and the same setup as during the discrimination training. Birds that preferred the unpalatable prey items in more than 4 trials were considered not having learned the prey discrimination successfully, and they were excluded from further testing. We set this criterion to ensure that all birds participating in the long-term memory test learned the discrimination similarly well, thus the potential differences in their performance could be explained by the treatment groups in the long-term memory test and not carried over from the discrimination training. Altogether, 3 birds were excluded because of this criterion. We also excluded a fourth bird as it showed a consistent preference for the cyan-colored paper baits both during the preference test and during the discrimination training.

The long-term memory test was conducted 4 wk later. Birds were divided into 3 treatment groups, balanced with respect to sex and age. The coloration of palatable baits (cyan without pattern) was the same as in the discrimination training and identical for all treatment groups. The coloration of unpalatable baits was different for each of the treatment groups: (1) the first group was tested with the same magenta-colored baits bearing black pattern as in the discrimination training (training-stimulus group), (2) the second group was tested with magenta-colored baits without black pattern (color-only group), and (3) the third group was tested with cyan-colored baits bearing black pattern (pattern-only group; Fig. 1). The second memory test consisted of 10 trials, each lasting for 4 min. In each trial, we recorded whether the bird handled one or both prey items, which prey item was handled first, latency of handling, and whether the bird ate (or attempted to eat) the pieces of mealworms. If the birds did not touch any of the prey items during a trial, it was repeated after 5 to 10 min.

All trials were recorded using Observer XT 8.0 © Noldus and a video camera.

### Data analyses

To assess the effect of the initial color preference on learning, we calculated a preference score (number of trials with preference for the cyan-colored bait in the preference test) for each bird and tested whether the initial preference for differently colored baits affected the speed of learning (measured as the number of trials needed to reach 5 correct decisions in a row) and the total number of correct decisions during the discrimination training. We considered a decision to be correct if the bird handled the palatable bait first, with handling being defined as touching the prey with the beak so that the birds’ decisions were based on visual cues alone.

Within-group comparisons were used to compare the performance of birds between the first 10 trials and the last 10 trials of the discrimination training, the next-day memory test, and the long-term memory test. We used the phase of the experiment as a predictor and tested its effect on the following response variables: the number of correct decisions (palatable bait handled first), the number of errors (unpalatable bait handled), the number of trials the birds attempted to eat the unpalatable bait (i.e. pecked the piece of mealworm with the beak), and the birds’ reaction toward the baits in the first trial of each experimental phase, i.e. whether the palatable bait was handled first, whether the unpalatable bait was handled at all, and whether the birds attempted to eat the unpalatable bait.

Between-group comparisons were performed to find out if there were differences among the 3 treatment groups in their performance in the long-term memory test. We used the treatment group as a predictor to compare the number of correct decisions, the number of errors, and the birds’ reaction toward the baits in the first trial of the long-term memory test, i.e. whether the palatable bait was handled first, whether the unpalatable bait was handled at all, and whether the birds attempted to eat the unpalatable bait. Furthermore, we compared the number of correct decisions and the number of errors during the next-day memory test using the treatment group as a predictor to find out if the differences in performance were already present during the next-day memory test or they can be explained by the different treatment groups in the long-term memory test.

We also assessed the differences in performance between males and females, and between yearlings and older adults in the preference test, in the discrimination training, in the next-day memory test, and in the long-term memory test. Using sex and age as predictors, we compared different response variables for each phase of the experiment. For the preference test, we compared the first preferred color and the number of trials where the cyan-colored bait was preferred. For the discrimination training, we compared the speed of learning, the number of correct decisions, the number of errors, and the latency to handle the unpalatable bait in the first trial. For the next-day memory test, we compared the number of correct decisions, the number of errors, and the latency to handle the unpalatable bait in the first trial. For the long-term memory test, we compared the number of correct decisions, the number of errors, and the latency to handle the unpalatable bait in the first trial.

Shapiro-Wilk normality test was used to establish whether the dependent variables showed a normal distribution, and statistical tests were chosen accordingly. In case of normal distribution, 1-way ANOVA with Tukey multiple comparisons of means (95% family-wise confidence level) or Welch Two Sample *t*-test were used. Otherwise Kruskal–Wallis test or Friedman test were used, followed by pairwise comparisons using either Wilcoxon rank sum (between-group) test or Wilcoxon signed rank (within-group) test with continuity correction (*P* value adjustment method: Bonferroni). Statistics were calculated in R version 4.0.0 (R Core Team 2020).

### Ethical note

The Ethical Committee of the Faculty of Science of Charles University gave consent to carry out the experiments. The experimental methods are included in ethical approvals issued by the Environmental Department of Municipality of Prague (S-MHMP-83637/2014/OZP-VII-3/R-8/F and MHMP-199460/2019), Ministry of Agriculture (13060/2014-MZE-17214 and 37428/2019-MZE-18134), and Ministry of the Environment of the Czech Republic (42521/ENV/14-2268/630/14 and MZP/2019/630/1082). All the birds were ringed and released at the location of capture after the experiments. Considering the STRANGE framework (Webster and Rutz 2020), the sex and age ratios were balanced among the experimental groups to avoid potential sampling biases.

## Results

### Learning and memory tests

In the preference test, 16 birds preferred the cyan-colored baits and 21 birds preferred the magenta-colored baits with black pattern. We did not find any effect of the initial preference for differently colored baits either on the number of correct decisions made by birds during the discrimination training (Welch Two Sample *t*-test, *t* = 0.016, df = 34, *P* = 0.987) or on the speed of learning (Kruskal–Wallis, χ^2^ = 0.015, df = 1, *P* = 0.902).

**Fig. 2.**
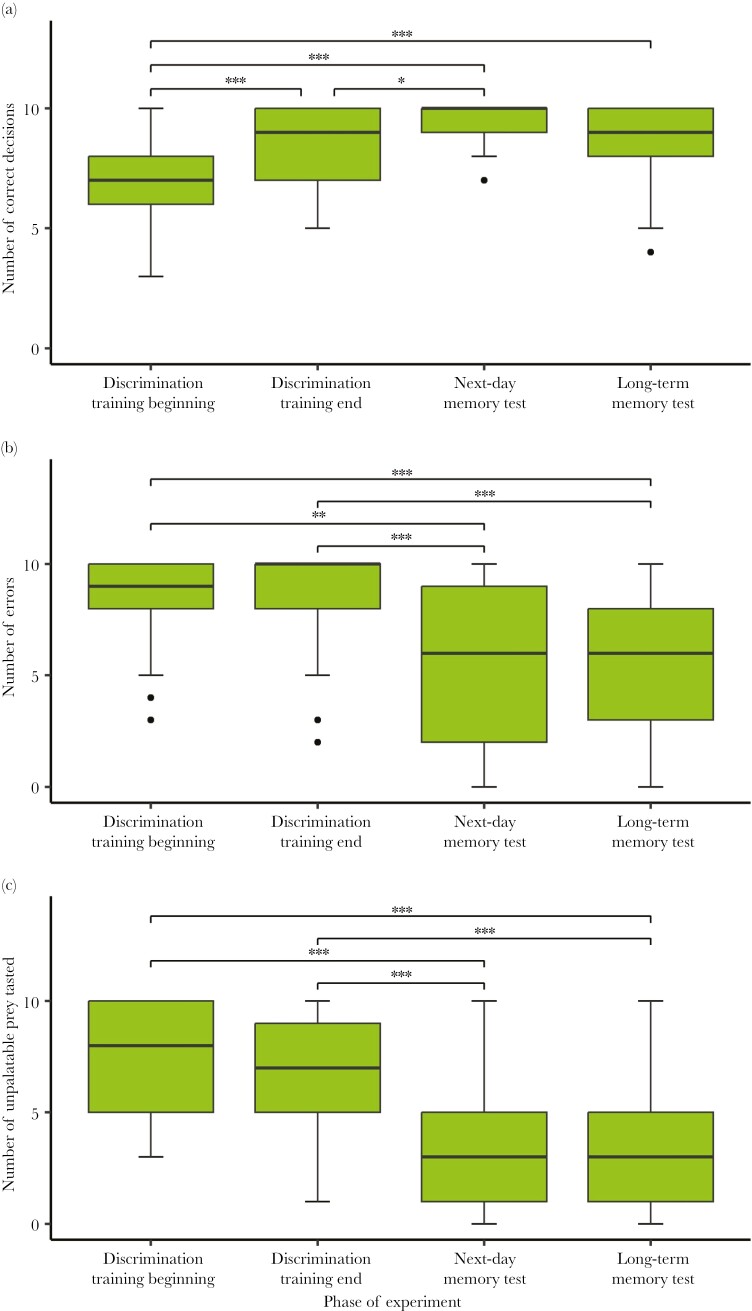
The performance of birds compared across the 4 phases of the experiment: the beginning and end of discrimination training, next-day memory test and long-term memory test. (a) The number of correct decisions; (b) the number of errors; (c) the number of trials where birds attempted to eat the unpalatable prey. The asterisks denote significance levels based on Wilcoxon signed rank test (*P < 0.05, **P < 0.01, ***P < 0.001). Horizontal lines represent medians, boxes are quartiles, vertical lines show minimum and maximum values, and dots represent outliers.

Comparison of the birds’ performance at the beginning of discrimination training (first 10 trials), the end of discrimination training (last 10 trials), and in both memory tests shows that the birds successfully learned to discriminate between the 2 differently colored baits and that they remembered their experience the next day and also after 4 wk. Comparing the performance of birds at the beginning and at the end of discrimination training, we found that the number of correct decisions was lower at the beginning of the discrimination training compared to the end of the training (Wilcoxon, *P* < 0.001; Fig. 2a); however, the number of errors (Wilcoxon, *P* = 1; Fig. 2b) and the number of trials where the birds attempted to eat the unpalatable prey (Wilcoxon, *P* = 0.6; Fig. 2c) did not differ between the 2 phases. The number of correct decisions was significantly different between the discrimination training and the memory tests (Friedman, χ^2^ = 59.931, df = 3, *P* < 0.001; Fig. 2a). The number of correct decisions was higher during the next-day memory test compared to both the beginning (Wilcoxon, *P* < 0.001) and the end of the discrimination training (Wilcoxon, *P* < 0.001). Performance in the next-day memory test tended to be better than the performance in the long-term memory test (Wilcoxon, *P* = 0.08), but the latter was still significantly better than the performance at the beginning of discrimination training (Wilcoxon, *P* < 0.001). The number of correct decisions was not different between the end of the discrimination training and the long-term memory test (Wilcoxon, *P* = 1). The number of errors was significantly lower during both memory tests compared to the beginning and the end of discrimination training (Friedman, χ^2^ = 41.923, df = 3, *P* < 0.001; Fig. 2b), but there was no difference between the next-day memory test and the long-term memory test (Wilcoxon, *P* = 1). We also found that birds attempted to eat the unpalatable prey less frequently during the memory tests compared to the beginning and the end of discrimination training (Friedman, χ^2^ = 65.836, df = 3, *P* < 0.001; Fig. 2c), but we did not find any difference between the 2 memory tests (Wilcoxon, *P* = 1). More birds handled the palatable baits first in the first trial of the next-day memory test compared to the first trial of discrimination training (Wilcoxon, *P* = 0.005), but there was no significant difference between the first trial of the next-day memory test and the first trial of the long-term memory test (Wilcoxon, *P* = 0.107) or between the first trial of discrimination training and the first trial of the long-term memory test (Wilcoxon, *P* = 0.624). The number of birds that handled the unpalatable bait in the first trial was not significantly different between the discrimination training and the memory tests (Friedman, χ^2^ = 4.571, df = 2, *P* = 0.102). To ensure that the differences in performance between the 3 treatment groups in the long-term memory test could not be attributed to potential differences carried over from discrimination training, we compared the performance of birds across the 3 treatment groups during the next-day memory test and we did not find any difference either in the number of correct decisions (Kruskal–Wallis, χ^2^ = 1.748, df = 2, *P* = 0.417) or in the number of errors (Kruskal–Wallis, χ^2^ = 3.644, df = 2, *P* = 0.162).

### Long-term memory test

**Fig. 3.**
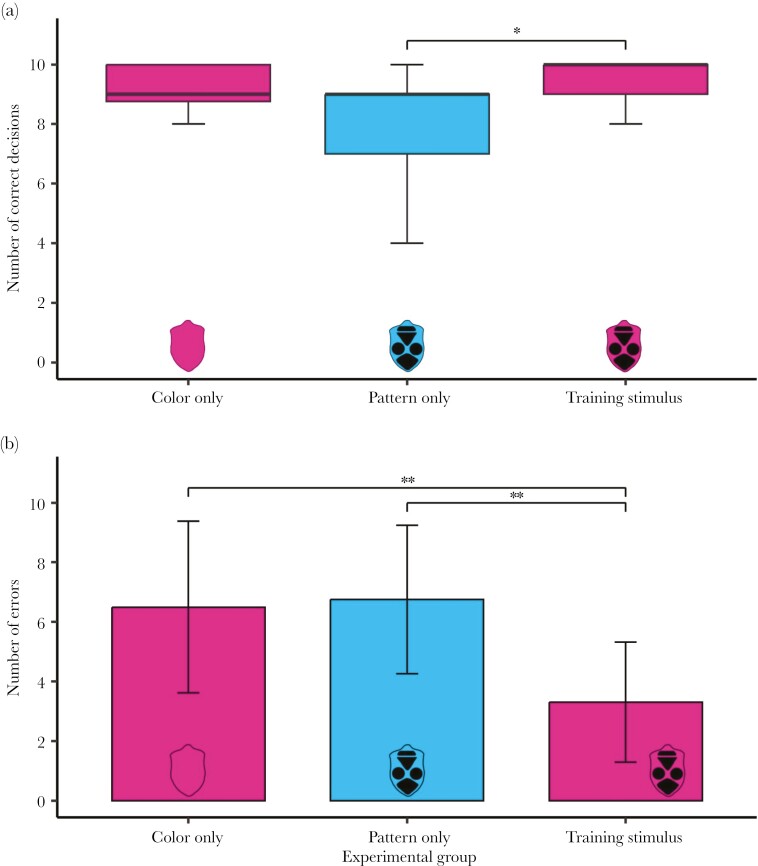
Comparison of the performance in the long-term memory test among the 3 treatment groups. (a) The number of correct decisions. The asterisk denotes the significance level based on Wilcoxon rank sum test (*P < 0.05). Horizontal lines represent medians, boxes are quartiles, and vertical lines show minimum and maximum values. (b) The number of errors. The asterisks denote significance levels based on 1-way ANOVA with Tukey multiple comparisons of means (**P < 0.01). Bars show means; vertical lines represent standard errors.

To find out which of the 2 warning signal components was more important for memory retention over 4 wk, we compared the performance of birds in the long-term memory test across the 3 treatment groups. We found a significant effect of the treatment group on the number of correct decisions (Kruskal–Wallis, χ^2^ = 7.893, df = 2, *P* = 0.019). Birds from the training-stimulus group made significantly more correct decisions than birds from the pattern-only group (Wilcoxon, *P* = 0.024; Fig. 3a). However, there was no difference either between the color-only and pattern-only groups (Wilcoxon, *P* = 0.194) or the color-only and training-stimulus groups (Wilcoxon, *P* = 1).

The number of errors was also significantly different among the 3 groups (ANOVA, *F* = 7.614, df = 2, *P* = 0.002; Fig. 3b). The training-stimulus group made significantly fewer errors compared to both the color-only (Tukey, *P* = 0.008) and the pattern-only groups (Tukey, *P* = 0.004). The number of birds that handled the palatable bait first in the first trial was not significantly different between the groups (Kruskal–Wallis, χ^2^ = 3.515, df = 2, *P* = 0.172). The number of birds handling the unpalatable bait in the first trial was also not different among the 3 groups (Kruskal–Wallis, χ^2^ = 1.675, df = 2, *P* = 0.432).

### Effect of age and sex

Yearlings and older adults did not differ in their initial preferences for differently colored baits. In the first trial of the preference test, 8 older adults and 6 yearlings chose cyan-colored baits, and 11 older adults and 12 yearlings chose magenta-colored baits with black pattern (Kruskal–Wallis, χ^2^ = 0.294, df = 1, *P* = 0.588). The number of trials where birds preferred the cyan-colored baits was also similar in the 2 age categories (Kruskal–Wallis, χ^2^ = 0.029, df = 1, *P* = 0.864). Yearlings tended to learn faster during the discrimination training, though this difference was not significant (Kruskal–Wallis, χ^2^ = 3.757, df = 1, *P* = 0.053). The number of correct decisions did not differ between the 2 age categories either during the discrimination training (Welch Two Sample *t*-test, *t* = −1.116, df = 34, *P* = 0.272) or during the next-day memory test (Kruskal–Wallis, χ^2^ = 0.051, df = 1, *P* = 0.821). The number of errors during the first ten trials of discrimination training (Kruskal–Wallis, χ^2^ = 0.363, df = 1, *P* = 0.547) and in the next-day memory test (Kruskal–Wallis, χ^2^ = 0.59, df = 1, *P* = 0.443) was also similar in the 2 age categories. However, the latencies to handle the unpalatable bait in the first trial of discrimination training were longer in older adults than in yearlings (Kruskal–Wallis, χ^2^ = 5.949, df = 1, *P* = 0.015). When comparing the performance of yearlings and older adults during the long-term memory test, we did not find any differences in the number of correct decisions (Kruskal–Wallis, χ^2^ = 0.497, df = 1, *P* = 0.481), the number of errors (Kruskal–Wallis, χ^2^ = 1.047, df = 1, *P* = 0.306) or in the latency to handle the unpalatable bait in the first trial (Kruskal–Wallis, χ^2^ = 0.433, df = 1, *P* = 0.511).

We did not find any significant differences between males and females in the number of correct decisions they made during discrimination training (Welch Two Sample *t*-test, *t* = 0.746, df = 33, *P* = 0.461) and during the next-day memory test (Kruskal–Wallis, χ^2^ = 1.845, df = 1, *P* = 0.174). The speed of learning (Kruskal–Wallis, χ^2^ = 0.302, df = 1, *P* = 0.583) and the number of errors made by males and females during the first 10 trials of discrimination training (Kruskal–Wallis, χ^2^ = 2.173, df = 1, *P* = 0.140) and during the next-day memory test (Kruskal–Wallis, χ^2^ = 0.125, df = 1, *P* = 0.724) also did not differ. The latency to handle the unpalatable bait in the first trial of discrimination training was also similar in males and females (Kruskal–Wallis, χ^2^ = 0.554, df = 1, *P* = 0.46). The 2 sexes also did not differ in their performance during the long-term memory test. Namely, the number of correct decisions (Kruskal–Wallis, χ^2^ = 0.083, df = 1, *P* = 0.77), the number of errors (Kruskal–Wallis, χ^2^ = 0.067, df = 1, *P* = 0.8), and the latency to handle the unpalatable bait in the first trial (Kruskal–Wallis, χ^2^ = 0.002, df = 1, *P* = 0.96) were similar between males and females.

## Discussion

Great tits in our experiment learned to discriminate between palatable and unpalatable prey differing in both color and pattern and remembered their experience even after 4 wk without any further reinforcement. The long-term memory test revealed that the number of correct choices between the 2 prey types offered in the memory test was similarly high in both the “training stimulus” and the “color only” groups; only the “pattern only” group performed worse. This indicates that color alone was sufficient for the birds to discriminate between the palatable and unpalatable prey. Furthermore, since both the palatable and unpalatable prey items presented to the “pattern only” group in the long-term memory test were cyan-colored, the birds might have made their choice solely based on the color ignoring the pattern, thus color might have overshadowed the presence of the pattern. The absence of any differences among the treatment groups in the next-day memory test indicates, that the worse performance of the pattern-only group reflects prioritizing the color cues in the prey choice, rather than potential differences carried over from discrimination training. These results are in line with the previous studies showing that color is the most salient cue for birds in prey discrimination (Osorio et al. 1999; Aronsson and Gamberale-Stille 2008; Rönkä et al. 2018; Kuklová et al. 2023). Furthermore, Kazemi et al. (2014) found that blue tits (*Cyanistes caeruleus*), preferentially use the color of the prey during discrimination learning and generalization and only attend to the pattern and shape of the prey when color does not convey information about prey palatability. The authors argue that high-salience traits (such as color) could overshadow other traits as blue tits avoided imperfect mimics if they shared the same color with models whereas mimics resembling models in shape or pattern did not gain protection. This phenomenon could contribute to the evolution of imperfect mimicry (Kazemi et al. 2014). Considering our results, it is therefore possible that the birds learned to associate the cyan color with prey palatability, and the birds tested in the pattern-only group might subsequently ignore the black pattern when deciding which prey to attack first.

Interestingly, the birds in the “training stimulus” group showed the lowest number of errors compared to both the “color only” and the “pattern only” group in the long-term memory test. This result indicates that birds were most successful in avoiding the unpalatable prey when they could use both color and pattern for its recognition after 4 wk, thus neither color nor pattern alone was sufficient to prevent the birds from attacking the unpalatable prey. This result appears to be in contrast to some of the previous studies, which show that, as the most salient cue, color may overshadow the pattern in prey discrimination by birds (Aronsson and Gamberale-Stille 2008). However, if color does not convey enough information about prey palatability, birds can use the presence of the pattern as an additional discriminative cue in a hierarchical way (Aronsson and Gamberale-Stille 2012). This is in accordance with our results because when the birds were choosing between the 2 prey types in the long-term memory test, they did so according to color, but when they were deciding whether to attack the potentially unpalatable prey, they paid attention to the presence of the pattern as well. Furthermore, Rowe et al. (2004) found that Müllerian (defended) mimics benefit from their similarity even if they share only their conspicuousness, and the similarity in pattern therefore does not seem to be important in early stages of predator discrimination learning. However, they argue that predators may pay more attention to other prey traits—such as pattern—at later stages of the learning process. This could at least partially explain the difference between our results and those of Aronsson and Gamberale-Stille (2008). Namely, in our experiment, the birds participated in a long discrimination training with only 2 prey items presented simultaneously, thus they had more opportunity to attend to other components of the warning signal in addition to color, which might contribute to fewer recognition errors in the memory test several weeks later. Another explanation could be that in the study by Aronsson and Gamberale-Stille (2008), the generalization test was carried out immediately after the last discrimination learning session, whereas in our study, the long-term memory test was preceded by the next-day memory test and conducted 4 wk after the discrimination training, thus the birds might have had more opportunity to consolidate their memories. Therefore, it is possible that color plays a dominant role especially in short-term memory, but both color and pattern cues can be retained over a longer time period and may play a role in prey recognition afterwards.

Taken together, we found that both color and pattern played a significant role when birds recognized an unpalatable prey after a long time period, although color seemed to be more important for making the first choice between 2 prey items. Color alone was sufficient when the birds decided which of the 2 prey types to attack first, but both color and pattern were retained in the long-term memory and might decrease the risk connected with handling defended prey.

Even though the birds’ age category did not affect their performance in discrimination training and memory tests, older adults took longer to attack the unpalatable prey in the first trial of avoidance learning compared to yearlings. Although there is a possible explanation for this result—older adults could be more experienced and might have generalized their previous experience with aposematic prey in the wild—it was not reflected in the preference test as older adults did not show any initial bias against the patterned prey. Contrary to our results, Lindström et al. (1999b) found that great tit (*P. major*) yearlings were more cautious toward black-and yellow painted mealworms compared to both older adults and hand-raised juveniles. Similarly, Exnerová et al. (2006) showed that great tit yearlings were more cautious than older birds when handling aposematic firebugs (*Pyrrhocoris apterus*), but this difference was not present in 3 other small passerine species: blue tits (*Cyanistes caeruleus*), robins (*Erithacus rubecula*), and blackcaps (*Sylvia atricapilla*).

Studies testing long-term memory retention of prey warning signals by predators are scarce with only a few including a memory test carried out several days or weeks after the discrimination training. Johnston and Burne (2008) showed that aposematic coloration (black and yellow stripes) is a more salient cue for domestic chicks (*Gallus domesticus*) than black or yellow coloration alone and the chicks remember their experience 24 h after a single aversive encounter with the prey. Skelhorn and Rowe (2006) trained domestic chicks to avoid red-colored prey with different levels of unpalatability and found that chicks learned to avoid more unpalatable prey faster and also remembered the experience better 48 h after the discrimination training. In a study testing adult great tits (*P. major*), Ham et al. (2006) found that typical warning colors (red and/or yellow) did not enhance memory retention—these colors were remembered equally well as gray in the memory test conducted 1 wk after the discrimination training. Furthermore, agamid lizards (*Diploderma swinhonis*) were found to remember red-colored unpalatable prey better than the green-colored one in the memory test following 60 d after a single aversive experience (Ko et al. 2020). Bogale et al. (2012) tested jungle crows (*Corvus macrorhynchos*) in a 2-choice color discrimination task (red, yellow, green, and blue) and found that most of the tested birds remembered the rewarded color 10 mo after the discrimination training.

Thus, although the evidence for the long-term memory retention of prey discrimination in great tits is not unique among visually oriented predators tested so far, our results indicate that predators can remember prey warning signals for at least a month and that multiple signal components may play an important role in subsequent prey recognition. This might be important for the evolution and maintenance of aposematic coloration, in particular in temperate regions, where the predators frequently do not have any contact with aposematic prey for several weeks or months. In future studies, it would be interesting to test how other components of visual warning signals, such as pattern contrast and body shape but also signals from other modalities, such as odors and sounds are remembered over a long time period.

## Data Availability

Analyses reported in this article can be reproduced using the data provided by Szabó et al. (2024).

## References

[CIT0001] Aoki M , Izawa EI, Koga K, Yanagihara S, Matsushima T. 2000. Accurate visual memory of colors in controlling the pecking behavior of quail chicks. Zool Sci. 17:1053–1059. https://doi.org/10.2108/zsj.17.1053 18522458

[CIT0002] Aronsson M , Gamberale-Stille G. 2008. Domestic chicks primarily attend to colour, not pattern, when learning an aposematic coloration. Anim Behav. 75:417–423. https://doi.org/10.1016/j.anbehav.2007.05.006

[CIT0003] Aronsson M , Gamberale-Stille G. 2009. Importance of internal pattern contrast and contrast against the background in aposematic signals. Behav Ecol. 20:1356–1362. https://doi.org/10.1093/beheco/arp141

[CIT0004] Aronsson M , Gamberale-Stille G. 2012. Colour and pattern similarity in mimicry: evidence for a hierarchical discriminative learning of different components. Anim Behav. 84:881–887. https://doi.org/10.1016/j.anbehav.2012.07.011

[CIT0005] Aronsson M , Gamberale-Stille G. 2013. Evidence of signaling benefits to contrasting internal color boundaries in warning coloration. Behav Ecol. 24:349–354. https://doi.org/10.1093/beheco/ars170

[CIT0006] Bogale BA , Sugawara S, Sakano K, Tsuda S, Sugita S. 2012. Long-term memory of color stimuli in the jungle crow (*Corvus macrorhynchos*). Anim Cogn. 15:285–291. https://doi.org/10.1007/s10071-011-0439-9 21792628

[CIT0007] Dolenska M , Nedvěd O, Veselý P, Tesařová M, Fuchs R. 2009. What constitutes optical warning signals of ladybirds (Coleoptera: Coccinellidae) towards bird predators: colour, pattern, or general look? Biol J Linn Soc. 98:234–242. https://doi.org/10.1111/j.1095-8312.2009.01277.x

[CIT0008] Exnerová A , Svádová K, Štys P, Barcalová S, Landová E, Prokopova M, Socha R. 2006. Importance of colour in the reaction of passerine predators to aposematic prey: experiments with mutants of *Pyrrhocoris apterus* (Heteroptera). Biol J Linn Soc. 88:143–153. https://doi.org/10.1111/j.1095-8312.2006.00611.x

[CIT0009] Gamberale-Stille G. 2001. Benefit by contrast: an experiment with live aposematic prey. Behav Ecol. 12:768–772. https://doi.org/10.1093/beheco/12.6.768

[CIT0010] Gamberale-Stille G , Tullberg BS. 1999. Experienced chicks show biased avoidance of stronger signals: an experiment with natural colour variation in live aposematic prey. Evol Ecol. 13:579–589. https://doi.org/10.1023/a:1006741626575

[CIT0011] Guilford T. 1986. How do “warning colours” work? Conspicuousness may reduce recognition errors in experienced predators. Anim Behav. 34:286–288. https://doi.org/10.1016/0003-3472(86)90034-5

[CIT0012] Ham AD , Ihalainen E, Lindström L, Mappes J. 2006. Does colour matter? The importance of colour in avoidance learning, memorability and generalisation. Behav Ecol Sociobiol. 60:482–491. https://doi.org/10.1007/s00265-006-0190-4

[CIT0013] Hauglund K , Hagen SB, Lampe HM. 2006. Responses of domestic chicks (*Gallus gallus domesticus*) to multimodal aposematic signals. Behav Ecol. 17:392–398. https://doi.org/10.1093/beheco/arj038

[CIT0014] Hunter JS. 2009. Familiarity breeds contempt: effects of striped skunk color, shape, and abundance on wild carnivore behavior. Behav Ecol. 20:1315–1322. https://doi.org/10.1093/beheco/arp144

[CIT0015] Johnston AN , Burne TH. 2008. Aposematic colouration enhances memory formation in domestic chicks trained in a weak passive avoidance learning paradigm. Brain Res Bull. 76:313–316. https://doi.org/10.1016/j.brainresbull.2008.02.016 18498948

[CIT0016] Kazemi B , Gamberale-Stille G, Tullberg BS, Leimar O. 2014. Stimulus salience as an explanation for imperfect mimicry. Curr Biol. 24:965–969. https://doi.org/10.1016/j.cub.2014.02.061 24726157

[CIT0017] Kikuchi DW , Allen WL, Arbuckle K, Aubier TG, Briolat ES, Burdfield‐Steel ER, Exnerová A. 2023. The evolution and ecology of multiple antipredator defences. J Evol Biol. 36:975–991. https://doi.org/10.1111/jeb.14192 37363877

[CIT0018] Ko YW , Liao CP, Clark RW, Hsu JY, Tseng HY, Huang WS. 2020. Aposematic coloration of prey enhances memory retention in an agamid lizard. Anim Behav. 161:1–13. https://doi.org/10.1016/j.anbehav.2019.12.015

[CIT0019] Kuklová L , Jůnová L, Kišelová M, Kuncová A, Exnerová A. 2023. Does the type of task affect prey discrimination learning in avian predators? Ethology. 129:527–540. https://doi.org/10.1111/eth.13390

[CIT0038] Lindström L , Alatalo RV, Mappes J, Riipi M, Vertainen L. 1999a. Can aposematic signals evolve by gradual change?. Nature 397(6716):249–251. https://doi.org/10.1038/16692.

[CIT0020] Lindström L , Alatalo RV, Mappes J. 1999b. Reactions of hand reared and wild-caught predators towards warningly coloured, gregarious and conspicuous prey. Behav Ecol. 10:317–322. https://doi.org/10.1093/beheco/10.3.317

[CIT0021] Osorio D , Jones CD, Vorobyev M. 1999. Accurate memory for colour but not pattern contrast in chicks. Curr Biol. 9:199–202. https://doi.org/10.1016/s0960-9822(99)80089-x 10074430

[CIT0022] Penacchio O , Halpin CG, Cuthill IC, Lovell PG, Wheelwright M, Skelhorn J, Harris JM. 2024. A computational neuroscience framework for quantifying warning signals. Methods Ecol Evol. 15:103–116. https://doi.org/10.1111/2041-210X.14268

[CIT0023] Prudic KL , Skemp AK, Papaj DR. 2007. Aposematic coloration, luminance contrast, and the benefits of conspicuousness. Behav Ecol. 18:41–46. https://doi.org/10.1093/beheco/arl046

[CIT0024] R Core Team 2020. R: A language and environment for statistical computing. Vienna, Austria: R Foundation for Statistical Computing. https://www.R-project.org/

[CIT0025] Rönkä K , De Pasqual C, Mappes J, Gordon S, Rojas B. 2018. Colour alone matters: no predator generalization among morphs of an aposematic moth. Anim Behav. 135:153–163. https://doi.org/10.1016/j.anbehav.2017.11.015

[CIT0026] Rowe C. 1999. Receiver psychology and the evolution of multicomponent signals. Anim Behav. 58:921–931. https://doi.org/10.1006/anbe.1999.1242 10564594

[CIT0027] Rowe C , Guilford T. 1999. The evolution of multimodal warning displays. Evol Ecol. 13:655–671. https://doi.org/10.1023/a:1011021630244

[CIT0028] Rowe C , Halpin C. 2013. Why are warning displays multimodal? Behav Ecol Sociobiol. 67:1425–1439. https://doi.org/10.1007/s00265-013-1515-8

[CIT0029] Rowe C , Lindström L, Lyytinen A. 2004. The importance of pattern similarity between Müllerian mimics in predator avoidance learning. Proc Biol Sci. 271:407–413. https://doi.org/10.1098/rspb.2003.2615 15101700 PMC1691604

[CIT0030] Ruxton GD , Allen WL, Sherratt TN, Speed MP. 2018. Avoiding attack: the evolutionary ecology of crypsis, aposematism, and mimicry. 2nd ed. Oxford, UK: Oxford University Press.

[CIT0031] Sherratt TN , Beatty CD. 2003. The evolution of warning signals as reliable indicators of prey defense. Am Nat. 162:377–389. https://doi.org/10.1086/378047 14582002

[CIT0032] Sherratt TN , Holen OH. 2018. When should receivers follow multiple signal components? A closer look at the “flag” model. Behav Ecol. 29:e6–e8. https://doi.org/10.1093/beheco/ary043

[CIT0033] Skelhorn J , Rowe C. 2006. Prey palatability influences predator learning and memory. Anim Behav. 71:1111–1118. https://doi.org/10.1016/j.anbehav.2005.08.011

[CIT0034] Svádová K , Exnerová A, Štys P, Landova E, Valenta J, Fučíková A, Socha R. 2009. Role of different colours of aposematic insects in learning, memory and generalization of naïve bird predators. Anim Behav. 77:327–336. https://doi.org/10.1016/j.anbehav.2008.09.034

[CIT0035] Svensson L. 1992. Identification guide to European Passerines. 4th ed. UK: British Trust for Ornithology.

[CIT0036] Szabó A , Bělová M, Exnerová A. 2024. Data from: I remember you! Multicomponent warning signals and predator memory. Behav Ecol. https://doi.org/10.5061/dryad.m63xsj4cm PMC11629967 39664075

[CIT0037] Webster MM , Rutz C. 2020. How STRANGE are your study animals? Nature. 582:337–340. https://doi.org/10.1038/d41586-020-01751-5 32541916

